# Myocardial tissue characterization in Chagas’ heart disease by cardiovascular magnetic resonance

**DOI:** 10.1186/s12968-015-0200-7

**Published:** 2015-11-18

**Authors:** Jorge A. Torreão, Barbara M. Ianni, Charles Mady, Evandro Naia, Carlos H. Rassi, Cesar Nomura, José R. Parga, Luis F. Avila, José A. F. Ramires, Roberto Kalil-Filho, Carlos E. Rochitte

**Affiliations:** Heart Institute, InCor, University of Sao Paulo Medical School,Cardiovascular Magnetic Resonance andComputed Tomography Sector, Av. Dr. Enéas de Carvalho Aguiar, 44, Andar AB, Cerqueira César, São Paulo, SP 05403-000 Brazil; Santa Izabel Hospital, Santa Casa de Misericórdia da Bahia, Bahia, Brazil

**Keywords:** Chagas’ heart disease, Myocarditis, Fibrosis, Late gadolinium enhancement, T2 weighted image, Cardiovascular magnetic resonance

## Abstract

**Background:**

Chagas’ heart disease is an important public health problem in South America. Several aspects of the pathogenesis are not fully understood, especially in its subclinical phases. On pathology Chagas’ heart disease is characterized by chronic myocardial inflammation and extensive myocardial fibrosis. The latter has also been demonstrated by late gadolinium enhancement (LGE) by cardiovascular magnetic resonance (CMR). In three clinical phases of this disease, we sought to investigate the presence of LGE, myocardial increase in signal intensity in T2-weighted images (T2W) and in T1-weighted myocardial early gadolinium enhancement (MEGE), previously described CMR surrogates for myocardial fibrosis, myocardial edema and hyperemia, respectively.

**Methods:**

Fifty-four patients were analyzed. Sixteen patients with the indeterminate phase (IND), seventeen patients with the cardiac phase with no left ventricular systolic dysfunction (CPND), and twenty-one patients with the cardiac phase with left ventricular systolic dysfunction (CPD). All patients underwent 1.5 T CMR scan including LGE, T2W and MEGE image sequences to evaluate myocardial abnormalities.

**Results:**

Late gadolinium enhancement was present in 72.2 % of all patients, in 12.5 % of IND, 94.1 % of the CPND and 100 % of the CPD patients (*p* < 0.0001). Myocardial increase in signal intensity in T2-weighted images (T2W) was present in 77.8 % of all patients, in 31.3 % of the IND, 94.1 % of the CPND and 100 % of the CPD patients (*p* < 0.0001). T1-weighted myocardial early gadolinium enhancement (MEGE) was present in 73.8 % of all patients, in 25.0 % of the IND, 92.3 % of the CPND and 94.1 % of the CPD (*p* < 0.0001). A good correlation between LGE and T2W was observed (r = 0.72, and *p* < 0.001).

**Conclusions:**

Increase in T2-weighted (T2W) myocardial signal intensity and T1-weighted myocardial early gadolinium enhancement (MEGE) can be detected by CMR in patients throughout all phases of Chagas’ heart disease, including its subclinical presentation (IND). Moreover, those findings were parallel to myocardial fibrosis (LGE) in extent and location and also correlated with the degree of Chagas’ heart disease clinical severity. These findings contribute to further the knowledge on pathophysiology of Chagas’ heart disease, and might have therapeutic and prognostic usefulness in the future.

## Background

*Trypanosoma cruzi* infection is responsible for the Chagas’ disease (CD) [1] and it is still a public health problem in South America with an estimated of 13 % of Latin American population at risk of contracting this disease. With an annual incidence of 29,925 cases in 21 Latin American countries, this disease still affects approximately 5.7 million people [2], with an average of 12,500 deaths per year [3]. Chagas’ disease has a disease burden, measured by disability-adjusted life years that is 7.5 times higher than malaria, which makes it the parasitic disease with the highest disease burden in the Western Hemisphere [4, 5]. Chagas’ heart disease is the most serious complication of CD, striking approximately one-third of seropositive individuals and the leading cause of death from heart failure in Latin America. The asymptomatic phase can last for decades, corresponding to the clinical indeterminate phase (IND), until unknown triggers initiate the progression to arrhythmias and heart failure in a subset, which represents approximately one third of the patients.

Myocardial fibrosis has been described in some studies with advanced Chagas’ heart disease subjects, using histologic confirmation [6, 7]. *T. cruzi* DNA can be detected by polymerase chain reaction during the chronic phases of Chagas’ heart disease in association with inflammatory infiltrate [8–10]. The presence of *T. cruzi* in combination with moderate to severe inflammation was demonstrated with the technique of immunohistochemistry [11]. Myocardial fibrosis and inflammation (using the Dallas criteria) were also demonstrated in patients with Chagas’ disease by histology and correlated to post-contrast increasing in myocardial signal intensity by rudimentary MR imaging [12].

Myocardial fibrosis in Chagas’ heart disease has also been demonstrated by cardiovascular magnetic resonance (CMR) using late gadolinium enhancement (LGE) technique, by few studies [13–15]. Additionally, CMR has demonstrated to detect myocardial inflammatory activity by probing myocardial edema and myocardial hyperemia in acute viral myocarditis, using T2-weighted and T1- weighted myocardial early gadolinium enhancement image sequences [16–20], with histological confirmation. However, the use of these techniques was not yet reported in patients with Chagas’ heart disease. This study aims to investigate the presence of late gadolinium enhancement, myocardial hyper intensity of signal in T2-weighted sequence (T2W) and in T1-weighted myocardial early gadolinium enhancement sequence (MEGE). in patients with Chagas’ heart disease in three distinct and progressive stages of the disease’s natural history. Assuming that those findings correspond to myocardial fibrosis, edema and hyperemia, respectively.

## Methods

In this cross-sectional study, we enrolled 54 patients with Chagas’ heart disease to undergo CMR. Patients with serologic confirmed Chagas’ disease diagnosis were referred to the study from our cardiomyopathy outpatient clinic. Exclusion criteria were history of myocardial infarction or coronary artery disease, more than two risk factors for CAD or diabetes mellitus (for those patients without anatomical confirmation of coronary arteries free of significant stenosis, by invasive coronary angiography or by coronary computed tomography angiography), valvular heart disease, previous viral myocarditis, other phases of cardiomyopathy, creatinine clearance below 30 ml/min/1.72 m^2^ and a contraindication to perform CMR. All patients underwent a brief interview prior to the MRI exam, which included information on height, weight, medical history and previous exams. We enrolled three subgroups at different stages of disease progression based on a classification of Chagas’ disease and grouped by the outpatient clinic: 1) a group of 16 patients without evidence of cardiac involvement by ECG, chest radiography and echocardiography called indeterminate phase of Chagas’ disease (IND), 2) a group of 17 patients who had cardiac phase without left ventricular systolic dysfunction (CPND), determined by ejection fraction equal or superior to 55 % by a routine clinical echocardiography analysis and electrocardiographic abnormalities (right bundle branch block with left anterior hemiblock) or 3) a group of 21 patients with the cardiac phase with left ventricular systolic dysfunction determined by ejection fraction inferior to 55 % by echocardiography analysis (CPD). The use of echocardiography for the definition of the left ventricular dysfunction and for the classification of chagasic cardiopathy phase followed the recommendation of the current guidelines for Chagas heart disease [21]. All CMR scans and image acquisitions were uneventful. All patients signed written informed consent approved by our local ethic committee (Comissão de Ética para Análise de Projetos de Pesquisa (CAPPesq) do Hospital das Clínicas da Faculdade de Medicina da Universidade de São Paulo) under number 0054/11.

### CMR methods

CMR was performed in all patients with a Philips 1.5 T scanner (Achieva, Philips, Best, The Netherlands). Images were acquired and coupled to the ECG during breath-hold, at left ventricle long and short axis, in the exact same location in the different image sequences. This allowed a precise comparison between cardiac function and regional myocardial structure. Cine images were acquired using SSFP sequence with TR 3.5 ms, TE 1.5 ms, flip angle 60 °, receiver bandwidth ± 125 kHz, field of view (FOV) of 35 × 35 cm, 256 × 148 matrix, temporal resolution 35 ms, 8.0 mm slice thickness, with 2 mm gap between the slices.

The detection of T2W was performed with a triple inversion-recovery pulse fast spin-echo (FSE) and long TE (always TE > 70 ms), with breath-hold, in short axis view of the left ventricle, which was carefully prescribed in order to guarantee the presence of an adequate skeletal muscle area for reference. In this sequence, we used the volumetric body coil acquisition to guarantee volume homogeneity and avoid signal dropout, as usually occurs with the surface coils. The parameters were: TR 2RR interval, TE 80-120 ms, Echo train length 24 (ETL), TI 140 ms, slice thickness 10 mm, with no gap (keeping the same center slice as for cine and LGE sequences) field of view 34 × 38 cm, matrix 128 × 128. MEGE acquisitions were performed pre and immediately post-gadolinium injection, with the same scanner parameters and calibrations. We applied a spin-echo free breathing sequence, in axial plane tipped inferiorly towards the left to align with the inferior wall of the left ventricle, with no change in acquisition parameters before and immediately after intravenous injection of 0.1 mmol/kg gadolinium based contrast (gadoteric acid, Gd-DOTA, Guerbet Aulnay-Sous-Bois - France), at a 2 ml/sec rate, followed by 10 ml of saline. The sequence began immediately after injection and lasted 3 to 4 min; the images reflect a gadolinium enhancement for an average time of 2 min. The sequence parameters were the following: Field of view should be adjusted to include the left upper arm, echo train length 2 to 4, slice thickness of 8 to 10 mm, 128 y-lines, and four signals averages.

Following the acquisition of spin-echo images, an additional dose of gadolinium-based contrast (0.1 mmol/kg) was injected for LGE acquisitions, using a typical inversion-recovery prepared gradient-echo acquired 10 to 20 min after the contrast, with the following parameters: TR 7.1 ms, TE 3.1 ms, flip angle 20°, cardiac phases 1, views per segment 16 to 32, matrix 256 X 192, slice thickness 8 mm, gap between slices 2 mm and field of view 32 to 38 cm, inversion time 150 to 350 ms, receiver bandwidth 31.25 kHz, number of excitations 1, acquisition every other heart beat. Additionally, we performed another sequence as described above but with an inversion time TI of 600 ms for further characterization of cardiac thrombus.

### Data analysis

The tests were analyzed in workstation with the CMR-42 software version 4.0.3 (Calgary Circle Cardiovascular Imaging Inc., Canada). The analyses were performed by an experienced professional blinded to the clinical classification and LGE results, randomly assigned using the Lake Louise criteria for myocarditis by CMR [22]. End-systolic, end-diastolic LV volumes, and LVEF were measured applying Simpson’s method. Segmental LGE transmurality and myocardial function were scored using standard LV 17-segment model. The T2W were assessed qualitatively by visual analysis and assisted by a 2SD thresholding technique that could be used by the observer to define the segments involved by myocardial edema. Additionally, a quantitative analysis used the average signal intensity ratio of the heart muscle and skeletal muscle, (pectoral or biceps). Ratios above 1.9 indicate positivity for the presence of myocardial edema. Patients were considered positive for myocardial edema if they had at least one of the above criteria. The assessment of MEGE was performed by detecting myocardial signal intensity gain after gadolinium injection and comparing it to the gain occurred in skeletal muscle, all in the same images. A signal increase ratio superior to 4 was considered positive for MEGE. If skeletal muscle had excessive increase in signal, superior to 20 %, myositis was to be considered and, to avoid underestimation in the MEGE ratio, an absolute myocardial signal increase superior to 45 % was used as a positive criterion [17, 23].

Late gadolinium enhancement patterns were classified as subendocardial, midwall, subepicardial, or transmural. The analysis included the number of LV segments involved by late gadolinium enhancement and an assisted automatic quantitative analysis to estimate LGE mass by a thresholding technique with pixel signal intensity 3 standard deviation above the mean of normal myocardial considered as myocardial fibrosis. In Chagas cardiomyopathy, heterogeneous and patchy myocardial fibrosis is the most frequent pattern, which challenges the use of Full Width at Half Maximum (FWHM) technique, where it is crucial to define a clear homogenous myocardial fibrosis area as a reference. By our knowledge, no definition on ideal threshold for detecting myocardial fibrosis in LGE images of Chagas cardiomyopathy patients is available. The authors performed a pilot analysis and felt that 3 standard deviation (SD) cutoff above normal myocardial signal intensity had the best agreement with visual LGE in a range of Chagas cardiomyopathy severity, which led the authors to choose in this study the 3SD threshold instead of 5SD (used in some studies involving hypertrophic cardiomyopathy). Quantification of late enhancement in the left ventricular myocardium used CMR42 software (Circle CVI, Calgary Canada).

### Statistical analysis

Comparisons of normally distributed continuous variables were performed by the Student *t* test and one-way analysis of variance with Bonferroni test for multiple comparisons. The Fisher exact test was used for proportions comparisons. The nonparametric test for discrete variables and non-normal continuous variables was Kruskal-Wallis rank test. Normality was determined by Shapiro-Francia W’ test. Simple linear regression was used between the MF mass and LVEF, end-diastolic volume, and end-systolic volume. Stata 8.0 (Stata Corp., College Station, Texas) was used, and *p <* 0.05 (two-tailed) considered statistically significant. Given the exploratory nature of the study, formal calculations of sample size were not performed. Based on an article published by our group [13], which investigated myocardial fibrosis in 51 patients with similar characteristics, and considering the myocardial fibrosis and inflammation as part of the same pathophysiological process, we chose to include at least 50 patients.

## Results

We have analyzed 54 patients, and 47 (87 %) had coronary angiography indicating absence of obstructive lesions (invasive coronary catheterization or coronary CT clinically indicated), The seven remaining patients had no history for coronary artery disease or presence of more than one risk factor. The mean age was 55.53 ± 11.11 years. The minimum age was 35 years and maximum 84 years. The population had similar age within the three phases of the disease. Regarding gender distribution, our sample showed no significant difference, however, there is an unequal distribution in the disease severity between genders, men are in more advanced stages of disease than women and with more LGE and T2W (Tables 1 and 2).

**Table 1:** Demographic characteristics, clinical and functional assessment by CMR

	Clinical phases			Total	*p*
	Indeterminate	CPND	CPD		
	(n = 16)	(n = 17)	(n = 21)	(n = 54)	
Age	57.8 ± 11.9	54.3 ± 10.3	54.7 ± 11.3	55.5 ± 11.1	0.61
Male	3 (18.7 %)	7 (41.1 %)	16 (76.1 %)	26 (48.1 %)	0.02
FC NYHA = I	16 (100 %)	14 (82.4 %)	1 (4.8 %)	31 (57.4 %)	<0.001
FC NYHA > I	0 (0)	3 (17.6 %)	20 (95.2 %)	23 (42.6 %)	<0.001
RVEDVi (ml/m2)	67.7 ± 14.9	70.4 ± 12.2	78.0 ± 22.7	72.6 ± 18.0	0.19
RVESVi (ml/m2)	25.9^a^ ± 5.6	32.0 ± 9.1	40.8^a^ ± 21.7	33.6 ± 15.8	0.01
RVEF (%)	60.9^a^ ± 7.1	54.7 ± 7.9	49.2^a^ ± 12.8	54.4 ± 10.9	0.04
LVEDVi (ml/m2)	72.0^a^ ± 15.4	84.6^b^ ± 22.7	137.0^a, b^ ± 44.0	101.4 ± 42.6	<0.001
LVESVi (ml/m2)	24.9^a^ ± 6.3	39.7^b^ ± 19.5	90.4^a, b^ ± 42.3	55.0 ± 40.6	<0.001
LVEF (%)	65.0^a^ ± 5.7	54.0^a^ ± 12.0	35.8^a^ ± 10.8	50.2 ± 15.8	<0.001
Mass index(g/m2)	55.2^a^ ± 10.5	70.9 ± 15.8	82.2^a^ ± 27.6	70.6 ± 22.8	0.01

Data are expressed as mean ± SD for numeric variables or absolute and relative frequencies (%) for categorical variables

*CPND* cardiac phase without left ventricular systolic dysfunction, *CPD* cardiac phase with left ventricular systolic dysfunctional. *LV* left ventricle, *EF* ejection fraction, *EDV* end-diastolic volume, *ESV* end-systolic volume, *RV* right ventricle. *FC* functional class, *NYHA* New York Heart Association

Difference by post hoc analysis (^a, b^)

**Table 2:** LGE and T2W among clinical and functional characteristics

	LGE		*p*	T2W		*p*	Total
	No	Yes		No	Yes		
	(n = 15)	(n = 39)		(n = 14)	(n = 40)		(n = 54)
Age	57.6 ± 11.9	54.6 ± 10.7	NS	58.1 ± 12.7	54.6 ± 10.5	NS	55.5 ± 11.1
LVEDVi (ml/m2)	23.9 ± 6.1	68.1 ± 41.9	<0.001	24.1 ± 6.4	65.9 ± 42.0	<0.001	55.0 ± 40.6
LVEF (%)	66.4 ± 5.1	43.3 ± 13.5	<0.001	66.7 ± 4.7	44.4 ± 14.1	<0.001	50.2 ± 15.8

Data are expressed as mean ± SD for numeric variables or absolute and relative frequencies (%) for categorical variables

*LVEF* left ventricle ejection fraction, *LVIEDV* Left ventricle indexed end-diastolic volume, *FC* functional class, *NYHA* New York Heart Association

The average LVEF was 50.2 ± 15.8 %, (normal > 55 %). The CPND showed an average LVEF of 35.8 ± 10.8 %, CPD and the indeterminate phase had 54.0 ± 12.0 % and 65.0 ± 5.7 %, respectively (<0.001). All with statistical differences by post HOC analysis (Bonferronni). The indexed end diastolic volumes of the left ventricle were progressively increased, along with the severity of the clinical phase. CPD showed an average of 137.0 ± 44.0 ml/m2 (Table 1).

There was significant association between the amount of LGE, T2W and MEGE with the severity of the clinical phases (*p <* 0.001), functional class (*p <* 0.001), LVEF (*p <* 0.001), and left ventricular diastolic volume (*p <* 0.001) (Tables 1, 2 and 3).

**Table 3:** Presence of LGE, TW2 and MEGE among clinical classification, FC NYHA, gender, LVFE and LVEDVi categories

	LGE	TW2	MEGE
IND	(n = 16)	(n = 16)	(n = 12)
	2 (12,5 %)	3 (18.8 %)	3 (25 %)
	Mass (g): 0,85 ± 2,47^a^	Segments: 0.31 ± 0.87 ^a, b^	
CPND	(n = 17)	(n = 17)	(n = 13)
	16 (94,1 %)	16 (94,1 %)	12 (92.3 %)
	Mass (g): 13.00 ± 10.8 ^a^	Segments: 3.24 ± 2.3 ^a^	
CPD	(n = 21)	(n = 21)	(n = 17)
	21 (100 %)	21 (100 %)	16 (94.1 %)
	Mass (g): 25.00 ± 11.9 ^a^	Segments: 3.67 ± 1.82 ^b^	
*p*	<0.0001	<0.0001	<0.0001
LVEF > 55 %	(n = 24)	(n = 24)	(n = 19)
	9 (37.5 %)	10 (41.7 %)	8 (42,1 %)
	Mass (g): 2.62 g ± 4.19 ^a^	Segments: 0.92 ± 1.3 ^a, b^	
LVEF =30-55 %	(n = 18)	(n = 18)	(n = 17)
	18 (100 %)	18 (100 %)	16 (94,1 %)
	Mass (g): 19.60 g ± 11.6 ^a^	Segments: 3.94 ± 2.2^a^	
LVEF < 30 %	(n = 12)	(n = 12)	(n = 6)
	12 (100 %)	12 (100 %)	6 (100 %)
	Mass (g): 29.00 g ± 10.8 ^a^	Segments: 3.67 ± 1.8 ^b^	
*p*	<0.0001	<0.0001	<0.0001
LVEDVi < 85	(n = 22)	(n = 22)	(n = 17)
	10 (45.5 %)	12 (54.5 %)	9 (52,9 %)
LVEDVi = 85-135 ml/m2	(n = 20)	(n = 20)	(n = 19)
	17 (85.0 %)	16 (80.0 %)	16 (84,2 %)
LVEDVi > 135 ml/m2	(n = 12)	(n = 12)	(n = 6)
	12 (100 %)	12 (100 %)	6 (100 %)
*p*	<0.0001	<0.0001	<0.0001
NYHA FC = 1	(n = 31)	(n = 31)	(n = 25)
	16 (51,6 %)	17 (54,8 %)	15 (60,0 %)
NYHA FC > 1	(n = 23)	(n = 23)	(n = 17)
	23 (100 %)	23 (100 %)	16 (94,1 %)
*p*	<0.0001	<0.0001	<0.0001
Male	(n = 26)	(n = 26)	(n = 19)
	23 (88,4 %)	23 (88,4)	17 (89,4 %)
Female	(n = 28)	(n = 28)	(n = 23)
	16 (57.1 %)	17 (60.7 %)	14 (60.8 %)
*p*	<0.0001	<0.0001	<0.0001
Total	(N = 54)	(N = 54)	(N = 42)
	39 (72.2 %)	40 (74.0 %)	31 (73,8 %)

Data are expressed in absolute and relative (%) frequency

*LGE* Late Gadolinium Enhancement image, *TW2* T2 weighted image, *MEGE* Myocardial Early Gadolinium Enhancement image, *LVEF* left ventricular ejection fraction, *LVEDVi* Left ventricle indexed end-diastolic volume, *IND* Indeterminate, *CPND* Cardiac phase without left ventricular systolic dysfunction, *CPD* Cardiac phase with left ventricular systolic dysfunctional, *FC* Functional class, *NYHA* New York Heart Association

Difference by post hoc analysis (^a, b^)

### Late gadolinium enhancement

**Fig. 1:**
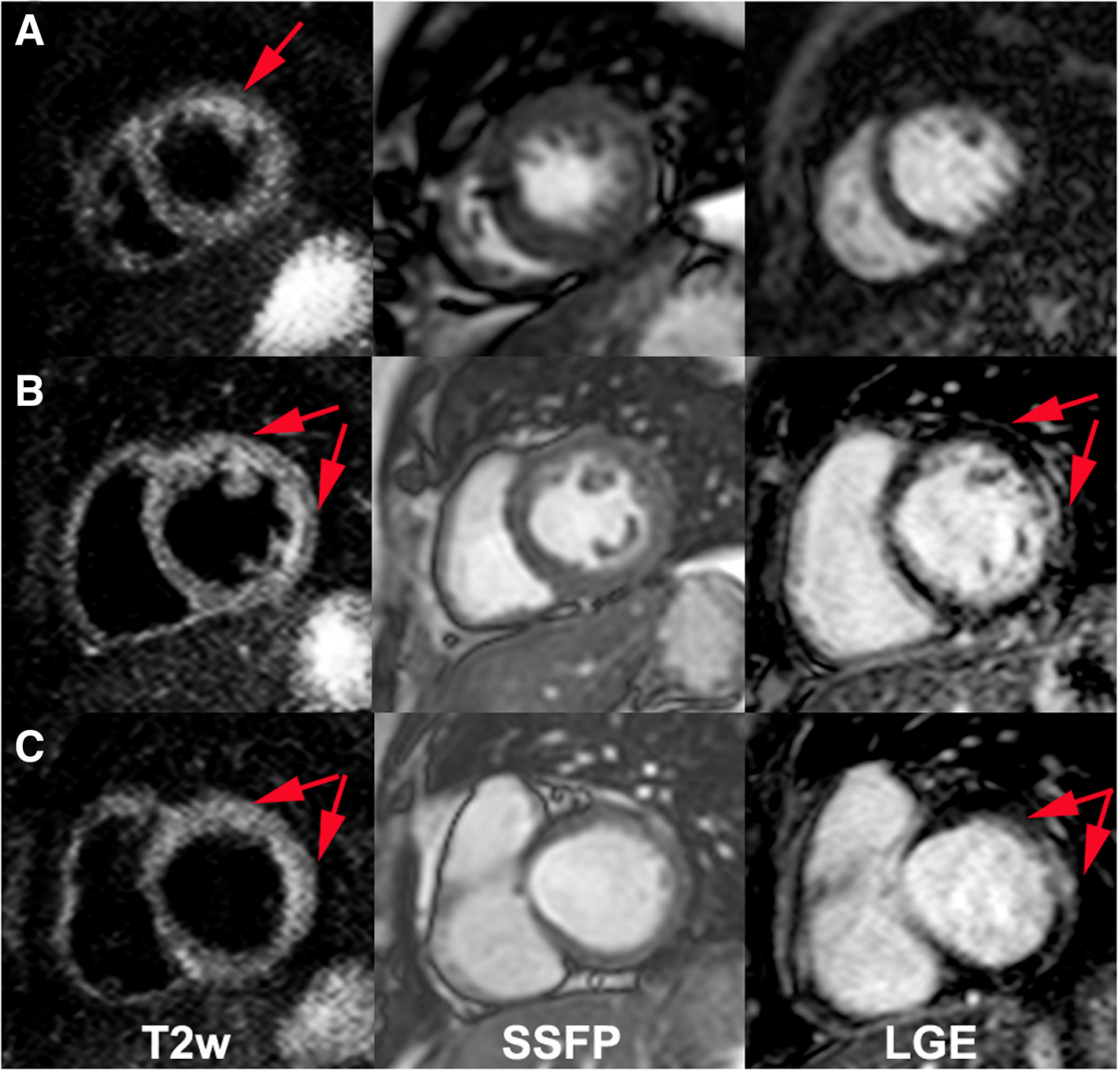
Apical (**a**), mid (**b**) and basal (**c**) short-axis images of a Chagas’ heart disease patient in the indeterminate phase (patient 54, IND) with T2w (*left column*), cine SSFP for anatomical reference (*mid column*) and LGE (*right column)*. *Red arrows* indicate increased myocardial signal intensity (T2W Ratio: 2.5). On the apical short-axis slice one can see a positive T2w image without correspondent LGE

**Fig. 2:**
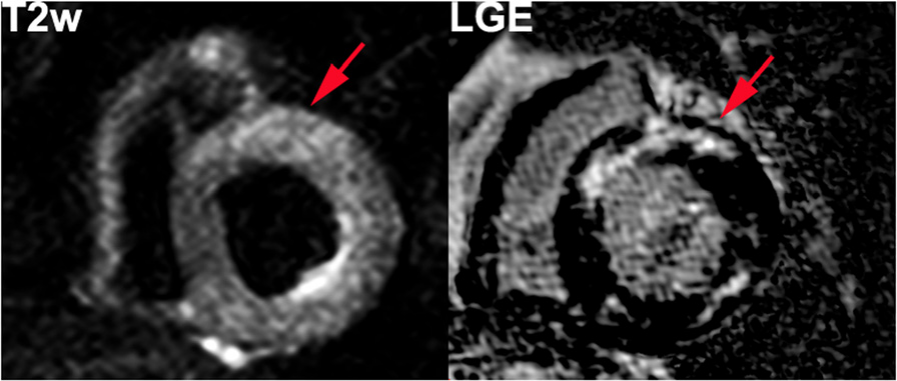
Short-axis images of a Chagas’ heart disease patient in the cardiac phase without LV dysfunction (patient 15, CPND). LGE (*right*) and T2 weighted (*left*) images with increased regional myocardial signal intensity on both techniques (T2W Ratio: 2.6)

**Fig. 3:**
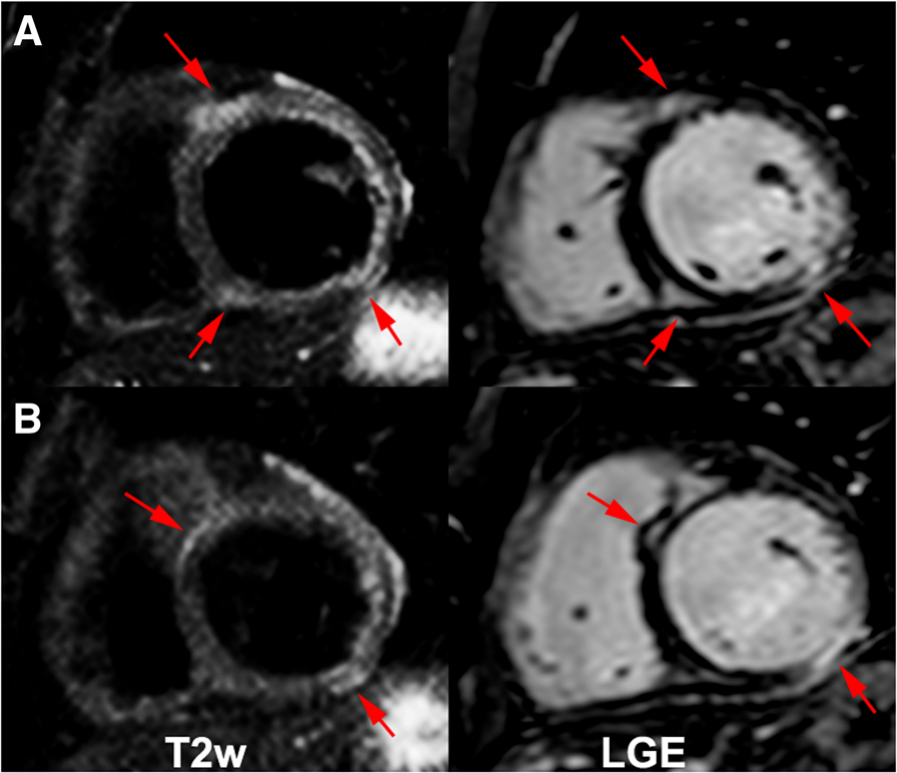
Mid (**a**) and basal (**b**) short-axis images of a Chagas’ heart disease patient in the cardiac phase with LV dysfunction (patient 24, CPD). LGE (*right*) and T2 weighted (*left*) images with increased regional myocardial signal intensity on both techniques (T2W Ratio: 2.4)

Present in 72.2 % of all patients. Two patients (12.5 %; mean MF mass of 0.85 ± 2.4 g) of the indeterminate phase (Fig. 1), sixteen patients (94.1 %; 13.0 ± 10.8 g) of the CPND (Fig. 2), and all patients of the CPD (Fig. 3), 21 patients; (25.2 ± 11.9 g; *p* < 0,0001) (Table 3).

### T2 weighted image

Present in 77.8 % of all patients. We have identified 3 (18.8 %) indeterminate phase (Fig. 1) patients (mean extent of 0.31 ± 0.87 segments). The CPND (Fig. 2) had TW2 in 94.1 %; with 3.24 ± 2.3 segments. All patients with CPD (Fig. 3) presented TW2 (3.67 ± 1.82 segments; *p* < 0,0001) (Table 3). Two patients had altered myocardial and skeletal muscle ratio (>1.9), although had no segmental T2w detected by the qualitative analyses and were considered positive (Patients 1 and 33).

### Myocardial early gadolinium enhancement

**Fig. 4:**
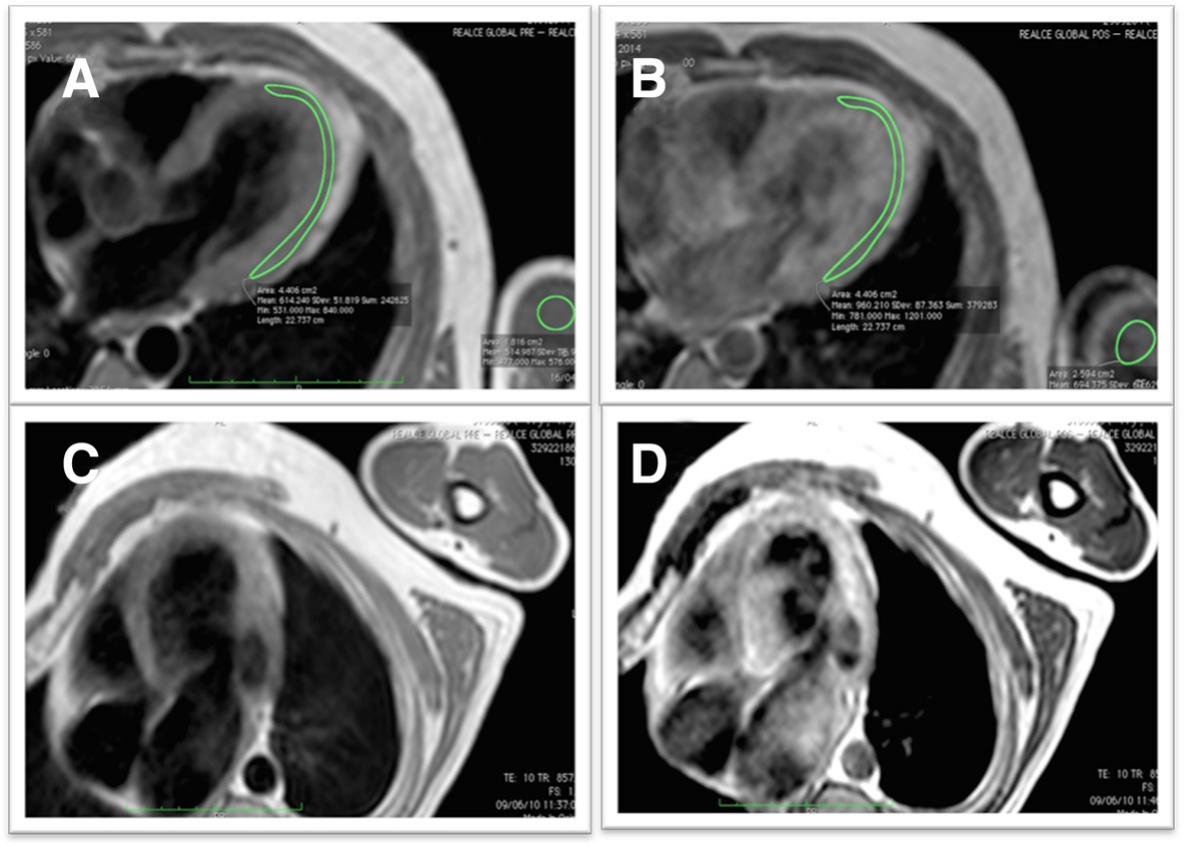
MEGE evaluation. Example of a negative case, pre contrast (**a**) and post contrast (**b**). Example of a positive case, pre contrast (**c**) and post contrast (**d**)

Forty-two patients underwent specified test. It was positive in 73.8 % of these patients. Three patients with MEGE (25 %) within the indeterminate phase, twelve (92.3 %) in CPND patients and sixteen (94.1 %) of the CPD patient group, *p* < 0,0001 (Table 3 and Fig. 4). Five patients had signal intensity increases in skeletal muscle, after contrast, greater than 20 %, considered suggestive of myositis (Patients 25, 28, 32, 41 and 48). A myocardial signal intensity increases after contrast superior to 45 % were considered positive in four individuals (Patients 25, 28, 32 and 41).

**Fig. 5:**
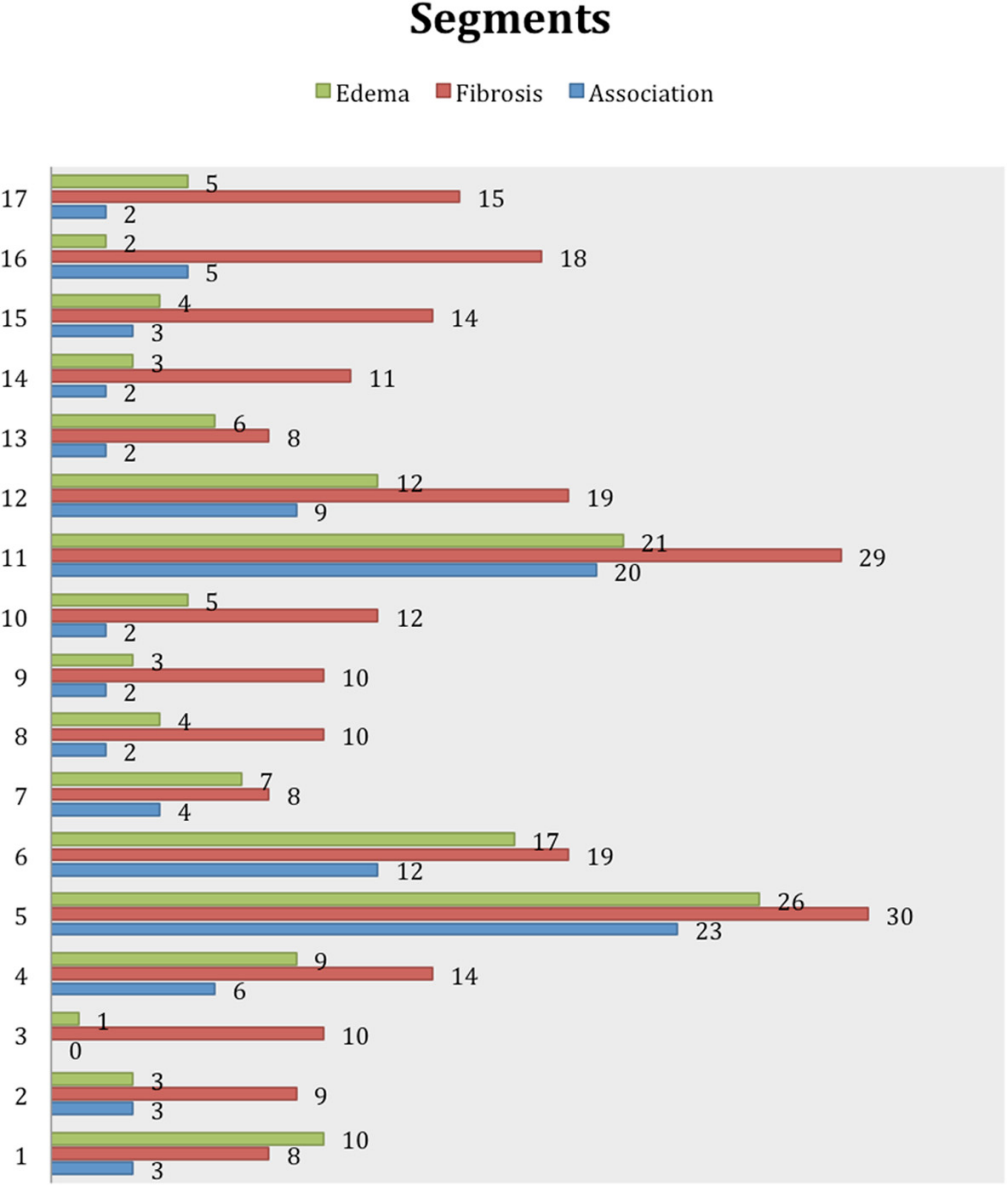
Association of segments with LGE and T2W positive, using the AHA segmentation model. Data are expressed as absolute frequency

**Fig. 6:**
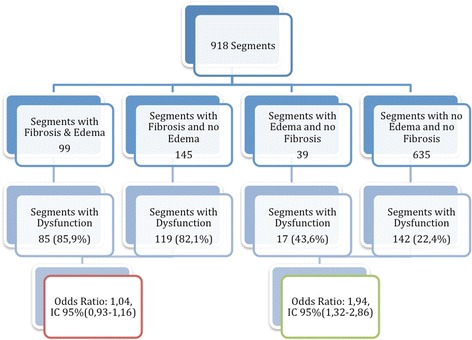
LV segmental dysfunction distribution based on the presence or absence of myocardial abnormalities. The presence of T2W increased the chance of segmental dysfunction in the absence of myocardial fibrosis

**Fig. 7:**
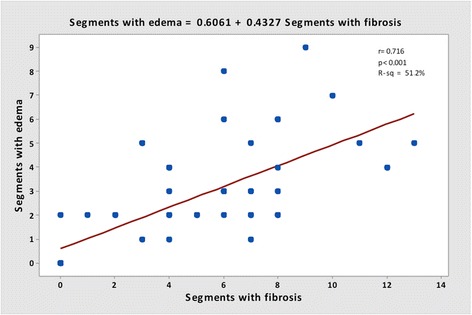
Correlation between segments with LGE and segments with T2W image

**Fig. 8:**
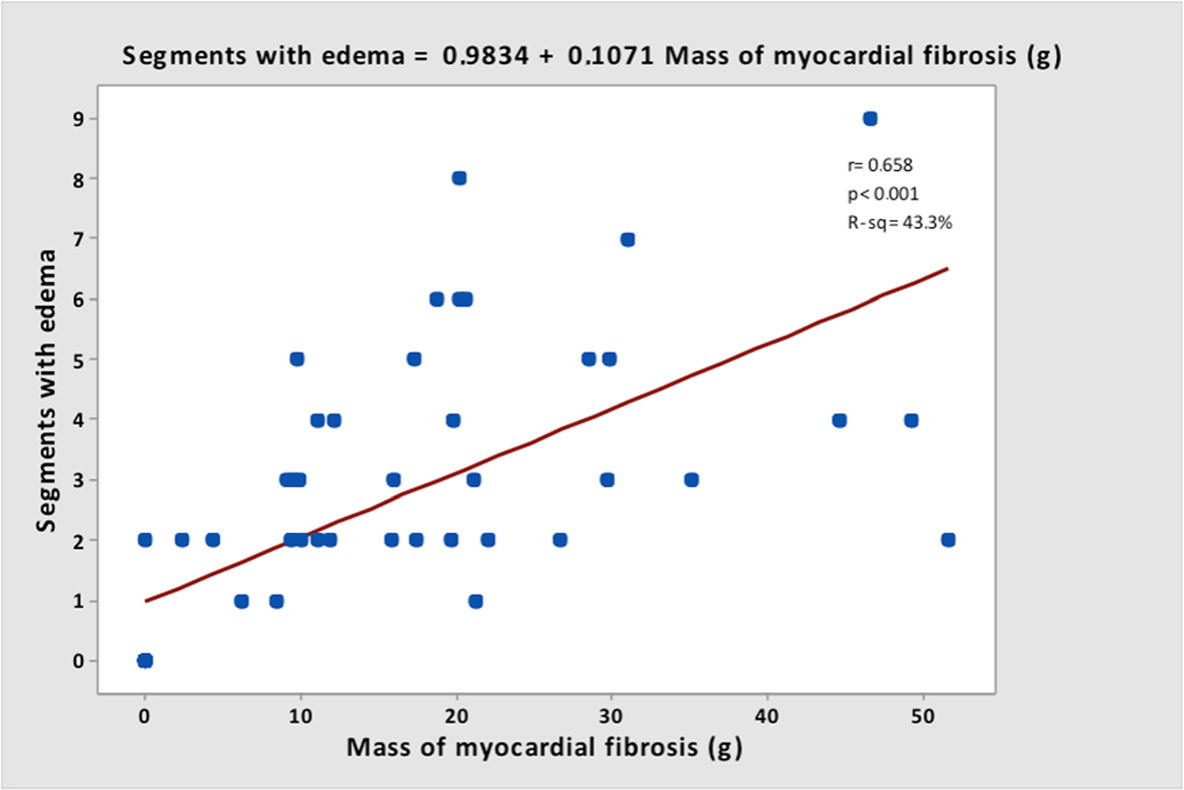
Correlation between mass of LGE and segments with T2W image

The segmental analysis was performed using the 17 segments AHA model, in 54 patients, totalizing 918 segments. Two hundred and forty-four segments (26.6 %) were identified with myocardial fibrosis, of which 204 (83.6 %) had segmental dysfunction. In 138 (15.0 %) segments T2W was detected, of which 102 (73.9 %) had segmental dysfunction. The coexistence of fibrosis and T2W was seen in 99 segments (10.8 % of all segments and 40.6 % of segments with myocardial fibrosis). Segmental dysfunction was detected in 363 segments. Presence of T2W did not perform as an independent factor to predict dysfunction when the segment had myocardial fibrosis, but it showed a significant chance for predicting segmental dysfunction within segments without myocardial fibrosis (Figs. 5 and 6). A good correlation between LGE and T2W was noticed (r = 0.72, and *p <* 0.001) (Figs. 7 and 8). All segments were involved in at least one patient, evidencing the diffuse nature of Chagas’ heart disease, although lateral and apical walls were more frequently affected.

## Discussion

CMR stands out amongst imaging techniques in the identification and quantification of myocardial fibrosis in Chagas’ heart disease, as has been described previously [13, 14]. The confirmation of myocardial fibrosis in patients in the indeterminate phase of the disease, contradicts the paradigm that this phase of the disease should not present any myocardial damage and place CMR as the current most sensitive technique to detect myocardial damage in Chagas’ heart disease. [24, 25]. It seems that patients within the indeterminate phase may have myocardial fibrosis and T2W in a such low degree that does not lead to myocardial dysfunction or remodeling and, therefore, may not be picked up by morphological and functional traditional methods, such as echocardiogram. On the other hand, CMR using tissue characterization techniques have been able to clearly detect these apparent small myocardial tissue abnormalities. This observation could also indicate that the concept of indeterminate phase may be outdated facing the new advances in cardiac imaging technologies such as CMR, and there may be an intermediate phases to those classically described.

This is the first study to investigate T2W and MEGE *in vivo* with a noninvasive tool, such as CMR, in patients with Chagas’ heart disease. The techniques applied in this study were well established in some important studies of myocarditis [16, 17, 19] and its diagnosis criteria were proposed in a landmark publication [22].

The natural history of indeterminate phase is to evolve to the cardiac phase in up to one third of this group or stay a benign condition without considerable deterioration of the myocardial status. Presence of positive criteria for T2W and MEGE in the indeterminate phase could have prognostic power to predict the evolution to the cardiac phase. The CPND is of great interest, considering that there is already a cardiac involvement, especially in the electric pathway, without significant systolic myocardial dysfunction. This phase may have also an unpredictable evolution and, therefore, it could become a valid maker for etiologic therapy, which is still controversial and awaiting for results of larger trials. Identifying myocardial abnormalities in phases of the disease with none or minor cardiac damage seems to be a reasonable alert to physicians of witch groups are at greater risk to develop systolic dysfunction and clinical heart failure. It also represents a major advance in understanding this disease, besides having therapeutic and prognostic importance.

The presence of myocardial abnormalities in patients with Chagas’ heart disease is not surprising and has been demonstrated on prior histological studies. Evidence suggests that parasites persist in the myocardium and the inflammatory and immune reactions are primarily responsible for the pathology. The presence of *T. cruzi* could be demonstrated using PCR and immunohistochemistry associated with inflammation [8–11]. Other studies have demonstrated common antigens between *T. cruzi* and human myocardial fibers, and they serve to sustain the autoimmune theory [26, 27]. This hypothesis states that chronic inflammation is perpetuated regardless of the presence of the parasite, as has been shown in some publications that used necropsy confirmation [28], and may be explained by the presence of cross-reactivity between these antigens and the immune system host. The presence of T2W in chronic phase of disease was related with severe impairment of myocardial status, and may represent the first *in vivo* CMR evidence that the inflammation of Chagas’ disease is perpetuated for an extended period after the acute phase.

Gutberlet et al. demonstrated the presence of persistent inflammation, confirmed by biopsy in 59 % of a group of patients with chronic viral myocarditis. This result differs of that found in this group of chronic Chagas myocarditis, where 74 % of the individuals had positive criteria for inflammation. This difference may be explained by the greater severity of myocardial damage found in chagasic myocarditis, where the mean LVEF was 50.2 % for all patients, 54.0 % for CP patients and 35,8 % for CPND patients, while in a chronic viral myocarditis group the LVEF was found within normal limits [19].

### Limitations

Our study represented the first to attempt an inflammatory characterization *in vivo* in patients with Chagas’ heart disease. Therefore we have faced some novel technical and clinical challenges. Our study had no control group, since we found concerning to enroll health subjects to contrast injection, however we used a very well established criteria for the detection of LGE, T2W and MEGE, with published historical controls. The edema and hyperemia criteria were extensively experimented on prior studies, compared with normal myocardial [16, 17, 19]. The analysis of T2W had a limitation on identifying segmental alteration in some of the cardiac cases due to LV wall deformation and thinning, especially in the more functionally impaired segments. The myocardium and skeletal muscle ratio criterion was of great use to correct discrepancies in those cases. Even though the analyses were made blinded to clinical classification and to LGE results, the readers had visual information on volumes and wall thickness encountered in the T2W sequence, what was considered, by the authors, also a limitation. Another important limitation was the lack of T1 and T2 mapping, once pulse sequences were not available in our site at the time of the acquisition. Arrhythmia data would be also useful clinical information in this scenario, but unfortunately our study was not designed to investigate VT presence or Rassi’s scores, which would require a Holter monitoring close to the CMR acquisition.

## Conclusions

CMR enables identification, location and quantification of myocardial fibrosis, T2W and MEGE on Chagas’ heart disease, adding potential information on disease severity. T2W and MEGE can be detected *in vivo* by CMR in patients within all clinical phases of Chagas’ heart disease, not only in the advanced phases but also in the early indeterminate phase. Moreover, those findings demonstrated a good biological correlation with the severity of Chagas’ heart disease, NYHA functional class, left ventricular ejection fraction, and with the presence of myocardial fibrosis.

## Abbreviations

CAD: Coronary artery disease
IP: Indeterminate phase group
CPND: Cardiac phase without left ventricular systolic dysfunctional
CPD: Cardiac phase with left ventricular systolic dysfunction
T2W: Myocardial hyper intensity of signal in T2-Weithed image
MEGE: T1-Weithed myocardial early gadolinium enhancement
LV: Left ventricular
LVEF: Left ventricular ejection fraction
LVEDVi: Left ventricle end-diastolic volume index
LVESVi: Left ventricle end-systolic volume index
LGE: Late gadolinium enhancement
MF: Myocardial fibrosis
CMR: Cardiovascular magnetic resonance
RT: Repetition time
ET: Echo time
NYHA: New York Heart Association
